# New tests of the distal speech rate effect: examining cross-linguistic generalization

**DOI:** 10.3389/fpsyg.2013.01002

**Published:** 2013-12-30

**Authors:** Laura C. Dilley, Tuuli H. Morrill, Elina Banzina

**Affiliations:** ^1^Department of Communicative Sciences and Disorders, Michigan State UniversityEast Lansing, MI, USA; ^2^Department of Psychology, Michigan State UniversityEast Lansing, MI, USA; ^3^Department of Communication Sciences and Disorders, Bowling Green State UniversityBowling Green, OH, USA

**Keywords:** distal speech rate, lexical perception, non-native perception, word segmentation, speech recognition

## Abstract

Recent findings [Dilley and Pitt, 2010. Psych. Science. 21, 1664–1670] have shown that manipulating context speech rate in English can cause entire syllables to disappear or appear perceptually. The current studies tested two rate-based explanations of this phenomenon while attempting to replicate and extend these findings to another language, Russian. In Experiment 1, native Russian speakers listened to Russian sentences which had been subjected to rate manipulations and performed a lexical report task. Experiment 2 investigated speech rate effects in cross-language speech perception; non-native speakers of Russian of both high and low proficiency were tested on the same Russian sentences as in Experiment 1. They decided between two lexical interpretations of a critical portion of the sentence, where one choice contained more phonological material than the other (e.g., /st
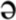
r
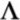
′na/ “side” vs. /str
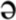
′na/ “country”). In both experiments, with native and non-native speakers of Russian, context speech rate and the relative duration of the critical sentence portion were found to influence the amount of phonological material perceived. The results support the generalized rate normalization hypothesis, according to which the content perceived in a spectrally ambiguous stretch of speech depends on the duration of that content relative to the surrounding speech, while showing that the findings of Dilley and Pitt (2010) extend to a variety of morphosyntactic contexts and a new language, Russian. Findings indicate that relative timing cues across an utterance can be critical to accurate lexical perception by both native and non-native speakers.

## Introduction

The process of recognizing words from continuous speech appears effortless most of the time. However, this apparent effortlessness obscures a great deal of cognitive complexity associated with two interrelated perceptual processes entailed in converting sound patterns into lexical representations. The first of these processes is word segmentation—that is, breaking up the continuous acoustic signal into candidate lexical items; few acoustic cues that consistently signal a word boundary have been identified, making this a difficult task (e.g., Cole and Jakimik, 1980). The second process is lexical access, which involves mapping chunks of sound onto stored lexical units in memory (See McQueen, 2006 for a review).

There are a number of theoretical proposals regarding how lexical access and word segmentation take place. Several accounts contend that speech input is parsed into a sequence of discrete and/or probabilistic inputs which generate patterns of lexical activation, as well as a string of lexical items, thereby achieving word segmentation (Marslen-Wilson and Welsh, 1978; McClelland and Elman, 1986; Norris and McQueen, 2008). A significant challenge faced by such approaches is in accounting for recognition of different acoustic-phonetic forms of a word, especially those produced with a casual speech style. It is well established that words can be produced with substantial phonetic variability. For example, casually-produced words may be significantly reduced (Barry and Andreeva, 2001; Ernestus et al., 2002; Johnson, 2004; Snoeren et al., 2008), and boundaries between words can be spectrally smeared due to coarticulation and other phonological processes (e.g., Shockey, 2003; Johnson, 2004). Explaining how listeners recover the lexical identity of words with such highly modified input is a challenging problem in spoken word recognition that underscores the need for further research.

Two broad categories of factors affect spoken word recognition, including recognition of casually spoken words. The first category consists of knowledge-based cues (e.g., semantics, syntax, lexical entries), which affect both word segmentation and lexical access (Dahan and Tanenhaus, 2004; Mattys et al., 2005). The second category consists of signal-based cues, including allophonic and coarticulatory information (Davis et al., 2002; Salverda et al., 2003; Mattys et al., 2005), phonotactic regularities (Vitevitch and Luce, 1999), and stress (Cutler and Norris, 1988; Mattys et al., 2005).

In the present paper, we investigate the signal-based cue of acoustic timing information and its role in lexical access and word segmentation of casual speech. Timing information in speech perception has been of substantial research interest over the years, both because of its role in perception of small, phoneme-sized units (e.g., Summerfield, 1981; Volaitis and Miller, 1992), as well as in perception of larger lexical structures (e.g., Davis et al., 2002; Salverda et al., 2003). Notably, a substantial body of work has shown that temporal information influences phoneme category boundaries and best exemplars in a variety of languages (Fujisaki et al., 1975; Nooteboom and Doodeman, 1980; Summerfield, 1981; Volaitis and Miller, 1992; Miller and Wayland, 1993; Sommers et al., 1994; Traunmüller and Krull, 2003). In addition, more recent work suggests that timing cues also influence lexical access for larger linguistic units (Davis et al., 2002; Salverda et al., 2003; Christophe et al., 2004; Niebuhr and Kohler, 2011; Reinisch et al., 2011). For example, Salverda et al. (2003) found that when listeners heard the syllable *ham*, which could be either the monosyllabic word “ham” or the first syllable of the word “hamster,” they were less likely to consider “hamster” as a lexical candidate when durational information matched the monosyllabic parse, “ham.”

In the aforementioned research, timing information influenced perception of segments or lexical structures, but in all cases listeners perceived a *fixed number* of segmental units (e.g., in specific words). Here, we build on novel, recent findings by Dilley and Pitt (2010) showing that manipulations of timing in speech context can cause listeners to perceive a *variable number* of segmental units for the same acoustic material. They investigated the role of speech rate in the perception of monosyllabic function words. In their Experiment 1 materials, function words were casually spoken and heavily coarticulated; For example “or” was spoken as [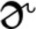] in the context of a preceding word ending in 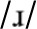 and a phrasal context where the function word was not syntactically obligatory (e.g., contexts such as *Don must see the harbor or boats*…). When the entire sentence fragment was presented in its unaltered form, listeners almost always heard the function word. However, when the speech rate of the context material prior to and following the vicinity of the function word was slowed down, function word reports dropped by more than half, from 79 to 33%, even though the region containing the function word was acoustically identical across conditions. Moreover, their Experiment 2 showed that when the context speech rate was speeded around a critical region of speech, listeners “hallucinated” hearing a function word that was never spoken.

Dilley and Pitt (2010) proposed a “generalized rate normalization” account of their findings, which built on prior work on speech rate normalization (Miller and Liberman, 1979; Pisoni et al., 1983; Sawusch and Newman, 2000) as well as dynamical systems theories of entrainment and rhythm perception (e.g., Povel and Essens, 1985; McAuley, 1995; Large and Jones, 1999; Saltzman and Byrd, 2000; Port, 2003). According to the generalized rate normalization account, the phonological content (e.g., number of words) perceived in a spectrally ambiguous stretch of speech depends on the duration of that content relative to the surrounding speech rate, as well as higher-level information, such as semantic and syntactic context.

The explanation put forward by Dilley and Pitt for their findings was that listeners failed to perceive the extra function word because the relative rate of the stretch of speech containing the function word was too fast relative to the surrounding context when the context was slowed down for that stretch to have contained the extra word (see Niebuhr and Kohler (2011) for a similar proposal). However, an alternative explanation was that listeners failed to perceive the extra word simply because there was a mismatch in speech rates between the context and the function-word-containing target; a rate mismatch could conceivably have interfered with detecting or attending to the extra word, particularly when it was heavily coarticulated. This alternative explanation is termed the *rate mismatch hypothesis*.

The first goal of the present paper was to provide a direct test of the rate mismatch hypothesis against the generalized rate normalization account of Dilley and Pitt's (2010)'s findings. According to the rate mismatch hypothesis, a rate mismatch that involved speeding up the context surrounding reduced phonological material should also reduce listeners' reporting of the coarticulated phonological material, relative to the original, matched rate. In contrast, the generalized rate normalization account predicts that only specific rate mismatch conditions should cause the phonological material to be missed, namely those for which the extra phonological material (e.g., the extra function word) was relatively fast compared with the surrounding speech rate.

A second goal of the present paper was to test whether and how the speech rate effects reported in Dilley and Pitt (2010) generalized across materials, listeners, and languages. In particular, we sought to determine whether the speech rate effects reported in Dilley and Pitt (2010) operate on a specific morphosyntactic class—i.e., function words—or instead on a specific prosodic class—i.e., reduced syllables. We hypothesized that the speech rate effects would generalize to the prosodic class of reduced syllables; if so, then effects of speech rate should be observed across items containing phonologically reduced syllables which have a wide range of morphosyntactic structures.

We also sought to test the generality of context speech rate effects on lexical perception through the use of materials from another language, namely, Russian. The combination of similarities and differences in the phonological systems of English and Russian made Russian a good choice for testing the linguistic generality of the speech rate effects first reported by Dilley and Pitt (2010) for English. Numerous phonological differences between the two languages—specifically, differences which were likely to affect speech timing and/or the acoustic-phonetics of casual speech—make the experiments using Russian materials a true test of generalization of Dilley and Pitt's (2010) findings. The most relevant of these differences for the current study is the distinctive system of vowel reduction in Russian. Whereas English features a binary contrast between reduced and unreduced (usually stressed) vowels, Russian features a three-way contrast between unreduced, moderately reduced, and extremely reduced vowels (Avanesov, 1956; Dauer, 1983; Crosswhite, 2000; Padgett and Tabain, 2005). Vowel reduction is phonetically realized through spectral changes, timing changes, and/or restrictions on the inventory of vowels in pre-stress and post-stress positions (Avanesov, 1956; Bondarko, 1998; Crosswhite, 2000; Padgett and Tabain, 2005). Thus, to successfully identify phonemes and lexical items in Russian, English native speakers need to be able to relate the combination of temporal and spectral information to three distinct vowel realizations in order to determine its level of reduction; this could be challenging, given that English only possesses only one type of reduced vowel.

We also sought to test the generality of context speech rate effects on lexical perception by contrasting the perceptions of timing and casual speech by (1) native Russian speakers with those of (2) English native speakers learning Russian. It is not at all clear that English native speakers learning Russian would be able to generalize upon their linguistic experiences in order to be able to glean appropriate timing cues from Russian casual speech to distinguish close lexical alternatives. Indeed, the many distinctive properties in the phonological systems of Russian and English make it challenging for native English speakers to perceive and produce timing and articulatory cues in Russian speech (Zsiga, 2003; Davidson, 2007, 2011). It is possible that the use of timing and casual speech segmental information by native English speakers learning Russian improves with substantial practice and experience, similar to other aspects of non-native phonology (Lively et al., 1993; Bradlow et al., 1997; Flege et al., 1997). If, on the other hand, learners of Russian can use temporal information from the speech context to distinguish lexical interpretations, even without explicit knowledge or training regarding such distinctions, this could indicate that timing information in speech is universally available to native and non-native speakers. In this case, rate normalization could occur automatically, even in second language speech perception. To test these possibilities, we contrasted the perceptions of native English speakers with low to moderate proficiency in Russian and those with substantial proficiency in Russian.

In order to address these issues, we conducted two experiments modeled after those of Dilley and Pitt (2010). We constructed sentences which contained a critical lexical sequence of one or more words which was phonologically similar but semantically unrelated to another lexical sequence with one less (reduced) syllable in Russian. Thus, the “Long” sequence /st
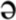
r
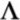
′na/ (“side”) had one more (reduced) syllable than the phonologically similar “Short” sequence /str
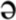
′na/ (“country”). Carrier sentences were constructed which were semantically congruent with both the Long and the Short interpretations of each lexical sequence. Long-Short lexical sequence pairs were morphosyntactically heterogeneous, but each Long version critically contained a phonologically reduced syllable, thereby permitting a test of whether the speech rate effects reported in Dilley and Pitt (2010) generalize to the prosodic class of reduced syllables.

Crucially in these experiments, timing was manipulated to include two kinds of conditions mismatching in rate: conditions in which the stretch of speech containing the target reduced syllable was relatively *long* compared with context, and conditions in which the stretch of speech containing the target reduced syllable was relatively *short* compared with context. If the rate mismatch account of Dilley and Pitt's reported speech rate effects on lexical perception is correct, then any rate mismatch should result in altered lexical perceptions. However, if the generalized rate normalization account proposed by Dilley and Pitt is correct, then only those rate mismatches in which the stretch of speech containing the target reduced syllable was relatively short compared with the context will produce altered lexical perceptions consisting of less phonological material. Experiment 1 addressed this question using a free-response task with native Russian speakers, and materials employing speech rate alterations similar to those used in Dilley and Pitt (2010). Experiment 2 tested the generality of lexical perceptions of Russian casual speech across three groups of participants: native Russian speakers, native English speaking learners of Russian with high proficiency in the language, and native English speaking learners of Russian with low to moderate proficiency in the language.

## Experiment 1

There were two goals of Experiment 1. The first was to specifically test whether the effects on lexical perception first demonstrated by Dilley and Pitt (2010) could be elicited by any rate mismatch between speech context and a target reduced syllable, or whether the proposed rate normalization hypothesis better accounts for the data. The central question was whether slowing down the speech rate around a critical region of speech (e.g., a portion containing /st
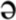
r
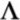
′na/ “side”) would result in Russian listeners hearing fewer phonological units for the same speech than when it was embedded in a context with a normal speech rate (so that listeners reported hearing e.g., /str
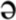
′na/ “country”, with one less syllable than in /st
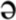
r
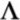
′na/ “side”). The second goal was to investigate the generality of the speech rate effects reported by Dilley and Pitt (2010) by replicating their findings with a language other than English, in this case, Russian.

### Method

#### Design

The experiment was conceived of as a 2 × 2 factorial design, with Rate Type (Compression vs. Expansion) and Locus (Target vs. Context) as the within-Subjects factors to create four Rate Type and Locus conditions. A separate control condition consisted of the Unaltered stimulus sentences, in which neither portion was manipulated, and the speech rate was matched across the sentence.

### Participants

The participants were 20 native Russian speakers residing in Latvia (11 male, 9 female), between the ages of 18 and 37, with self-reported normal hearing. Procedures were approved by the IRB of Michigan State University and Bowling Green State University.

#### Materials

Eighteen phonologically-related phrase pairs (e.g., “Short,” /str
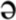
′na/ vs. “Long,” /st
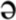
r
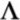
′na/) were identified (see Appendix, Table A1). Each “Long” version of a pair was embedded in a semantically valid sentence context, e.g.:

“
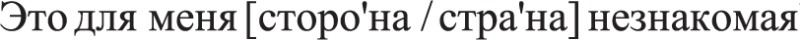
”. [et.
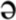

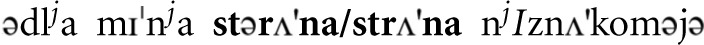
] (Translation: “This [side (of town)/ country] is unknown to me”).

Stimulus phrases were selected such that each “Long” version had a “Short” version consisting of less phonological material (e.g., one less syllable or one less word) than the “Long” versions. In other words, each “Long” version could perceived as the “Short” version and would still be grammatical. For instance, in the example above, the “Long” version consists of a three-syllable word, whereas the “Short” version consists of a phonologically-related two-syllable word. Of the 18 stimulus phrase pairs, 10 were such that the “Short” version contained one less syllable than a phonologically-related word in the “Long” version. Moreover, six were such that the “Short” version of the phrase contained one less word (i.e., one less monosyllabic function word with #CV# or #V# structure) than the “Long” version. Finally, for the remaining two phrase pairs, the “Short” version contained one less segment adjacent to a word boundary than the “Long” version; neither of these two cases involved a singleton vs. geminate contrast.

Sentences were recorded in Russian in a sound-attenuated booth by three native Russian speakers (2 male and 1 female), all graduate students from Bowling Green State University. Speakers were given a list of sentences containing the Long lexical sequences, as well as filler sentences, 244 sentences in total. Initial semantic context cues were added to the otherwise neutral sentences to ensure the correct sequence was read. Speakers were instructed to first read each sentence silently and then speak from memory twice. Instead of explicitly asking speakers to act naturally, casual speech productions were obtained by instructing talkers to speak from memory instead of reading, and placing experimental items strategically later in the long list, such that speakers became fatigued and less careful in their speech articulation. A single token of the Long sequence version of each sentence pair was selected as the basis for experimental items. Tokens were selected for which the critical Long sequence was judged to have been spoken casually and whose intonation patterns were deemed acceptable for both Long and Short phrase contexts.

Recorded sentences were then subjected to time manipulation using Praat software (Boersma and Weenink, 2002) by altering the duration of either the Target (the unstressed phoneme(s) that distinguish(es) the Long lexical sequence from the Short lexical sequence, plus one to two immediately surrounding phonemes, not more than 3 segments in total), or the Context (all sentence material before and after the Target). Target and Context portions were spliced out of original utterances, time-compressed by a factor of 0.6 or time-expanded by a factor of 1.9, and recombined. These compression and expansion factors were chosen to approximate the rates that had been used with English stimuli, which had been found to yield altered perception of the Target (Dilley and Pitt, 2010; Heffner et al., 2013). These rates had also been determined by trained phoneticians to be perceived as clearly faster or slower than the unaltered speech rate, while remaining intelligible with respect to linguistic content. Time compression and expansion of the stimuli was performed using the PSOLA (pitch-synchronous overlap-and-add) algorithm as implemented in Praat (Moulines and Charpentier, 1990; Moulines and Verhelst, 1995). This method of time scaling speech has been widely used in speech perception research (e.g., Dupoux and Green, 1997; Reinisch et al., 2011), due to the high quality of the resulting synthetic speech, including minimal acoustical distortions. In addition, special care was taken to prevent discontinuities at splicing points (i.e., zero crossings).

**Figure 1:**
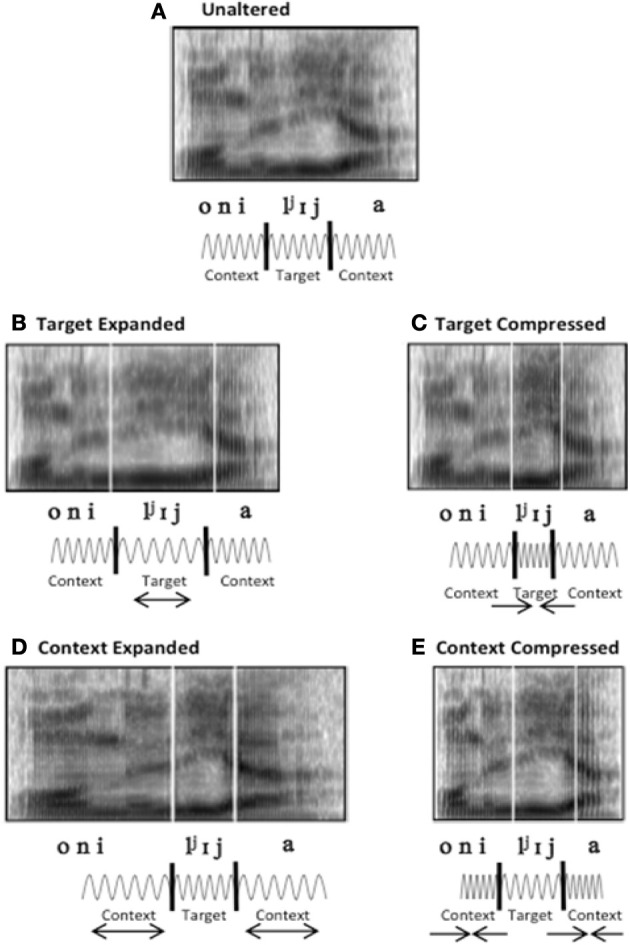
**Spectrograms of an example stimulus item for each of the Time Manipulation conditions. (A)** Represents the Unaltered condition. Vertical white lines in spectrograms delineate Target and Context portions of speech material. Arrows beneath schematic waveforms positioned below spectrograms indicate which portion of the stimulus item was subject to time alteration for Time Manipulation conditions **(B–E)**. Arrows pointing outward indicate time-expansion, while arrows pointing inward indicate time-compression.

The stimulus sentences were created using time-manipulated (Compressed or Expanded) Target or Context portions, to produce four experimental Time Manipulation conditions (see Figure 1). In each condition, the speech rates of the Target and Context were mismatched, such that some portion of the sentence was faster or slower than the original utterance due to the time manipulation. The combinations of Rate Type (Compressed and Expanded) and the Locus of the manipulation (Target and Context) yielded the following four conditions: (i) In the Target Compressed condition, the Target was time-compressed, while the Context rate was unaltered. Thus, the duration of Target was relatively short and the reduced syllable was predicted to be less likely to be perceived (i.e., fewer long lexical interpretations should be reported, despite the fact that it had occurred in the original production of the utterance). (ii) For the Context Expanded condition, the Context was time-expanded, while the Target rate was unaltered. In this condition, the duration of the Target was also relatively short compared to the Context, which was predicted to again reduce the likelihood of perceiving the reduced syllable (i.e., fewer long lexical interpretations should be obtained). (iii) For the Target Expanded condition, the Target was time-expanded, while the Context was unaltered in rate. The duration of the Target was thus relatively long, which we predicted would make the critical reduced syllable likely to be perceived (i.e., the proportion of long lexical interpretations would remain the same as in the Unaltered condition, or possibly increase). (iv) For the Context Compressed condition, the Context was time-compressed, while the Target was unaltered in rate. Again, the Target was relatively long compared to the Context, which was predicted to make the reduced syllable likely to be perceived (i.e., there should be no reduction in the proportion of long lexical interpretations). An Unaltered control condition was also created using the original recordings of the sentences, in which no rate change was imposed on either the Target or Context portion of the sentence. The speech rate of the Target and Context were matched, and the duration of the critical reduced syllable in the Target was expected to be perceptible, since it had been uttered as part of the long lexical sequence.

In sum, based on the hypotheses put forward in Dilley and Pitt (2010), we predicted a critical difference between certain Time Manipulation conditions and the Unaltered (baseline condition). Since the original recording of the utterance used in all of the Time Manipulation conditions contained a production of the Long lexical sequence, it is specifically the conditions in which the Target was relatively short compared to the Context (the Context Expanded and Target Compressed conditions) which should result in a reduction in the proportion of long lexical interpretations. Conversely, conditions in which the Target is relatively long compared to the Context (the Context Compressed and the Target Expanded Conditions) should not result in a reduction in the proportion of long lexical interpretations compared to the Unaltered condition; the proportion of long lexical interpretations should be similar or higher than in the Unaltered condition (if the Unaltered condition is not at ceiling, or 100%).

Filler sentences were constructed in the same way as the experimental items and contained one word that was phonologically similar to another semantically possible word. Approximately one-third of the filler items was temporally modified by time-compressing the entire item by a rate of 0.6, while another third was modified by time-expanding the entire item by a rate of 1.9, respectively. The remaining items were not altered in rate.

#### Procedure

Five lists were constructed from 18 experimental items and 22 filler items; the first five stimuli on the list were filler items, and the remaining items occurred in quasi-random order with the constraint that no more than three items of the same type (experimental or filler) occurred in a row. Each experimental item was presented only once on a list, with the pairing of experimental items and conditions counterbalanced across the five lists. Participants were randomly assigned to one of the five lists.

The experiment was presented via Praat software. Participants were seated in front of a computer with headphones on and given an answer sheet on which a series of sentences appeared, each with a blank space corresponding to the ambiguous portion of the phrase. Participants were instructed to click on a button on the computer screen to play a sound file; they then wrote down the word they heard corresponding to the blank in each sentence. Participants could listen to each sentence twice, and could proceed through trials at their own pace.

### Results

**Figure 2:**
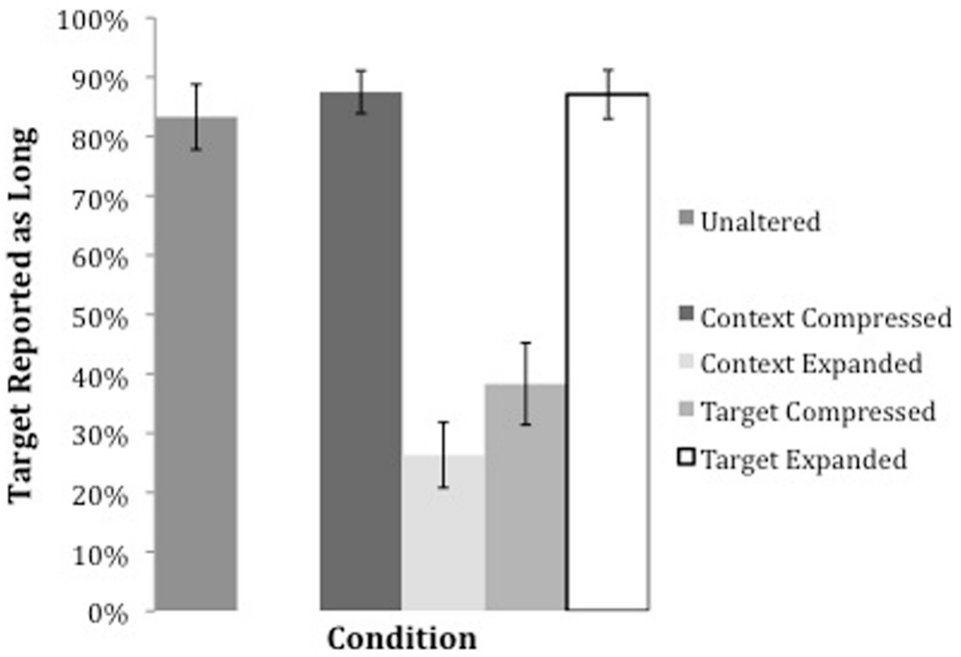
**Rate of “Long” responses to lexical sequences in each experimental stimulus in Experiment 1**.

Responses were coded for the proportions of “Long” responses to the lexical sequence. Figure 2 shows the proportion of Long responses for each Time Manipulation condition. In the Unaltered (control) condition, in which no change in rate was imposed on the stimuli, native speakers identified the naturally spoken stimuli as Long 83% of the time, showing that, at baseline, listeners perceived the veridical phonological material that had been spoken a high proportion of the time.

A logit mixed-effects model analysis (e.g., Jaeger, 2008) was performed in R (Bates et al., 2012) to examine the reliability of differences between the Time Manipulation conditions in the elicitation of a Long response. A model was fit with condition as a fixed effect and subjects and items as random effects. This model is presented in Table 1, with coefficient estimates, standard errors, Wald's *z-*values, and the significance level for each predictor. Treatment coding with the Unaltered (control) condition as the baseline was used to examine the contrast between the rate manipulated conditions and the Unaltered condition.

**Table 1:** **A log mixed-effects model with coefficient estimates, standard errors, Wald's *z-*values, and the significance level for each Time Manipulation condition**.

	**Estimate**	***SE***	***z*-value**	***p-*value**
(Intercept—*Unaltered*)	2.05	0.49	4.23	*****p* < 0.001****
Context expanded	−3.19	0.48	−6.60	***p* < 0.001**
Target compressed	−2.48	0.46	−5.38	*****p* < 0.001****
Context compressed	0.32	0.51	0.63	*p* = 0.53
Target expanded	0.75	0.56	1.34	*p* = 0.18

These results demonstrate that both the Context Expanded and Target Compressed conditions result in a significantly lower likelihood of eliciting a Long response than the Unaltered condition (β = − 0.19, *z* = −6.60, *p* < 0.001 for Context Expanded, and β = −2.48, *z* = −5.38, *p* < 0.001 for Target Compressed). As predicted, the conditions in which Long responses are less likely are those in which the Target is *relatively* short compared to the Context. In the conditions in which the Context was compressed or the Target itself was expanded, the likelihood of a Long response was slightly higher than in the Unaltered baseline condition (in which listeners had reported a Long response 83% of the time); however, these estimates were not significantly different from the Unaltered condition estimate.

To examine whether there was any independent effect of Rate Type or Locus of Manipulation, only the rate manipulated conditions were included in a model, without the Unaltered condition. An ANOVA to test whether Rate Type (Compressed and Expanded) or Locus (Context and Target) themselves were significant predictors in the model revealed that while neither Rate Type (*p* = 0.41) or Locus (*p* = 0.63) were significant predictors of Long responses, the predicted interaction between these two factors emerged (χ = 72.24, *p* < 0.001), indicating again that the Expanded Rate Type for the Context Locus and the Compressed Rate Type for the Target Locus resulted in lowered proportions of Long responses, and not a general effect of time manipulation itself. Thus, as indicated by the differences between the Time Manipulation conditions above, it is only the conditions in which the Target is relatively short which result in lowered proportions of Long responses.

### Discussion

The results of Experiment 1 show that the perception of reduced syllables depended on the existence of a specific temporal relationship holding between a word and the distal speech rate context in which that word occurs. According to the generalized rate normalization hypothesis proposed in Dilley and Pitt (2010), altered lexical perception should occur in those conditions in which the critical lexical item (the Target) was made to sound relatively *short* compared with the distal speech rate context, either by slowing down the context surrounding the word, or speeding up the word itself. In the present experiment, this occurred in the Target Compressed and Context Expanded conditions. Consistent with this prediction, listeners perceived the Target as relatively short precisely in the Target Compressed and Context Expanded conditions, as evidenced by lower likelihood of a Long response in these conditions. The results were furthermore inconsistent with the rate mismatch hypothesis, which predicted that all conditions in which the speech rate of the critical lexical item and the context were mismatched (i.e., Target Compressed, Target Expanded, Context Compressed and Context Expanded conditions) should have resulted in altered lexical perception. These findings thus clarify the nature of the distal speech rate phenomenon first reported by Dilley and Pitt (2010) by showing that not just any mismatch in speech rate across an utterance produces a change in lexical perception; this is consistent with the generalized rate normalization account.

The results reported here furthermore show that the speech rate effects originally reported in Dilley and Pitt (2010) apparently generalize to the prosodic class of reduced syllables, as opposed to operating on a particular morphosyntactic class (i.e., function words). The experimental stimuli in the present experiment contained a variety of morphosyntactic structures, including content words and polysyllabic words. Yet, the presence of a crossover interaction between the factors of Rate Type and Locus indicate that the rate of the critical lexical item (i.e., the Target) relative to the Context determined lexical perception for these varied structures.

Experiment 1 also provides the first replication in a different language of the speech rate phenomenon demonstrated for English by Dilley and Pitt (2010). Slowing down the context speech rate resulted in Russian listeners hearing fewer phonological units than were spoken in a critical region of speech, relative to the same speech embedded in a distal context presented at the unaltered spoken rate. These findings represent the first report of perception of casual speech in Russian and add to a growing body of research on perception of casual speech across languages (Barry and Andreeva, 2001; Ernestus et al., 2002; Mitterer and Ernestus, 2006; Snoeren et al., 2008).

Overall, this research adds to prior published findings (Dilley and Pitt, 2010; Heffner et al., 2012) that manipulating context speech rate can lead to the perception of variable numbers of phonological units (e.g., more or fewer syllables and/or words). Earlier research had shown that speech rate could affect perception and processing of a specific, fixed numbers of segmental units, either by affecting the perceived identities of those segments or the locations of phoneme boundaries and best exemplars along a continuum (Miller and Liberman, 1979; Volaitis and Miller, 1992; Sommers et al., 1994) or the speed and accuracy of lexical access for a fixed amount of phonological material (Davis et al., 2002; Salverda et al., 2003). The present results, and those of Dilley and Pitt (2010), are also important in illustrating that temporal manipulations to the context that is distal to the locus of perceptual change (non-adjacent phonemes, and in the case of monosyllabic function words, across a word boundary) can produce relatively large effects on listeners' perceptions of words.

## Experiment 2

Experiment 1 showed that native Russian listeners' perception of reduced syllables depends on the existence of specific relative timing relationships across portions of an utterance, replicating previous similar findings for English. Experiment 2 tests whether temporal information is perceived and accurately integrated in lexical recognition by learners of Russian, despite substantial differences between the English and Russian phonological systems (Crosswhite, 2000; Padgett and Tabain, 2005). Experiment 2 also investigates whether or not the use of timing information in non-native speech perception improves with increased language proficiency, using three groups of listeners: native Russian speakers, native English speakers with low proficiency in Russian, and native English speakers with high proficiency in Russian.

### Methods

#### Design

Experiment 2 used a 3 × 2 × 2 mixed factorial design, with Proficiency (Native, High-proficiency and Low-proficiency) as a between-subjects variable, and Rate Type (Compressed vs. Expanded) and sentence Locus (Target vs. Context) as within-subjects variables, leading to four Time Manipulation conditions, as in Experiment 1. A separate control condition consisted of the Unaltered stimulus sentences, also as in Experiment 1.

#### Participants

There were 44 participants in the experiment, all of whom were at least 18 years of age. The Native Russian-speaking group consisted of thirteen participants, all graduate students from Bowling Green State University and Michigan State University (5 male, 8 female), between the ages of 23 and 31. All of the native Russian speakers were born in Russia. The Low proficiency, non-native Russian-speaking group consisted of 17 native English speakers from Bowling Green State University, Michigan State University and the University of Michigan (5 male, 12 female), aged 19–45. All but one of the Low proficiency participants had 1–2 years of formal instruction in Russian, while the remaining participant had 2 years of experience living in a Russian speaking country, with active daily communication in Russian, but no formal instruction. The High proficiency, non-native Russian-speaking group consisted of 14 native English speakers from Bowling Green State University, Michigan State University, and the University of Michigan (6 male, 8 female), aged 19–53. These participants had either (i) formal instruction in Russian of no less than 4 years, and/or (ii) a minimum of 4 years living in a Russian-speaking country, and/or (iii) a minimum of 5 years of active daily communication in Russian with a Russian native speaker. Proficiency level was further assessed with a survey completed by participants, in which they reported information about their Russian skills and language use patterns. All of the High proficiency learners rated their speaking and listening skills as “fluent/excellent” or “very good” (on a 4-point scale in which “poor” was the lowest rating and “fluent/excellent” was the highest) while all of the Low proficiency learners rated their speaking and listening skill as “good” or “poor.” Care was taken to ensure that participants, especially low proficiency ones, were familiar with the vocabulary items: before the beginning of the experiment, participants were given ample time to silently read the list and report words anywhere in the sentence that appeared new or confusing, which were then translated and/or explained by the experimenter (e.g., “[s
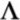
′rok] is a kind of bird”). No mention was made of timing information in clarifying terms. None of the participants was unfamiliar with more than three words in the lists. Procedures were approved by the IRB of Michigan State University and Bowling Green State University.

#### Materials

The stimuli for Experiment 2 were the same as in Experiment 1.

#### Procedure

In the experimental task, participants were presented with the two alternatives that existed for each sentence and had to choose which of the alternatives matched their perception. Each participant was presented with a list of 18 experimental sentences and 22 filler items. Five lists were created; an experimental sentence occurred on each list only once, either as the Unaltered version or in one of the time manipulated conditions. Experimental items were counterbalanced across the five lists and approximately equal numbers of participants heard each list. No experimental items from the same time manipulation condition occurred in immediate succession. Experimental items were interspersed with up to three filler sentences; the first five items in each list were always filler sentences.

The experiment was presented using Praat software (Boersma and Weenink, 2002). Participants were instructed to listen to sound files over headphones by clicking on a button on the computer screen to play the sound file, and then to circle one of the two options provided for each sentence on their answer sheets. Participants could listen to each sentence twice, and could proceed through trials at their own pace.

### Results

**Figure 3:**
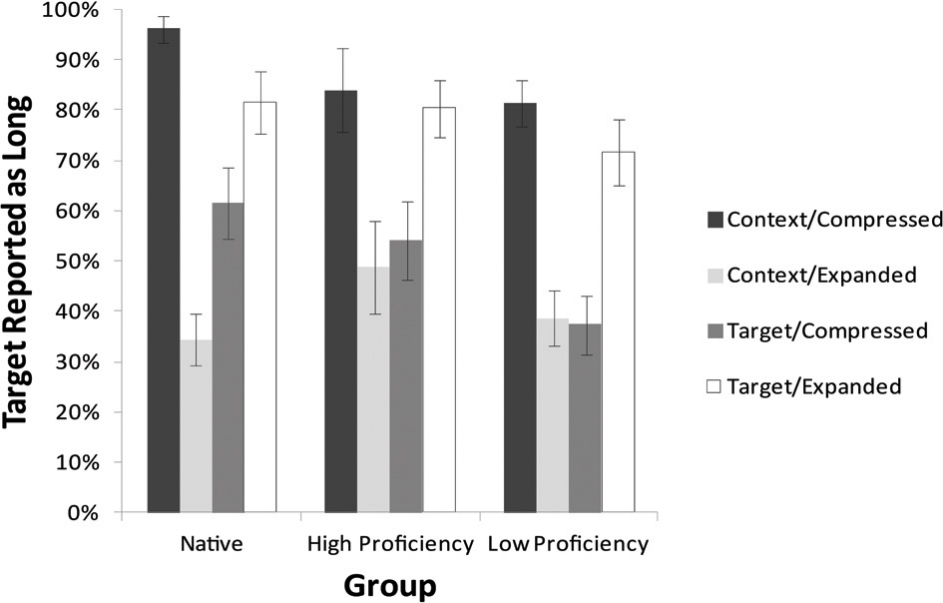
**Rate of “Long” responses to lexical sequences in each experimental stimulus for three Proficiency groups in the two-alternative task of Experiment 2**.

**Figure 4:**
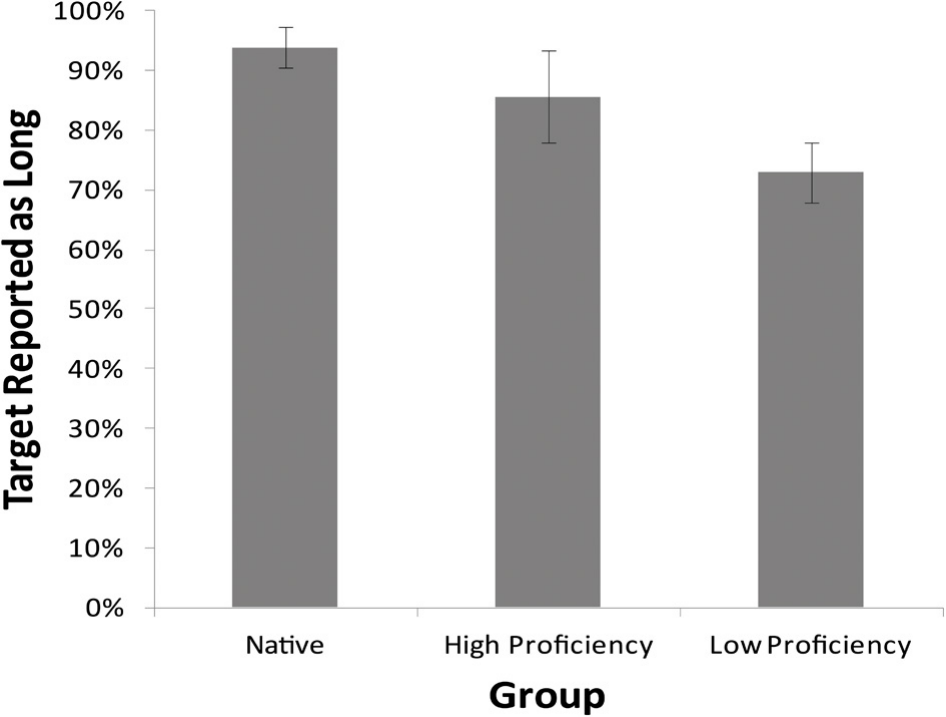
**Rate of “Long” responses to lexical sequences for three Proficiency groups in the Unaltered condition of the two-alternative task in Experiment 2**.

Figure 3 shows the proportion of “Long” responses for each timing manipulation condition for the three groups differing in Russian proficiency. Figure 4 shows the proportion of “Long” responses for the Unaltered condition. A logit mixed-effects model analysis was performed in R to examine the reliability of the effects of Time Manipulation condition and Proficiency on the likelihood of eliciting a Long response. A model was fit with Time Manipulation condition and Proficiency as a fixed effects and subjects and items as random effects. This model is presented in Table 2 with coefficient estimates, standard errors, Wald's *z-*values, and the significance level for each predictor. The intercept represents the Unaltered condition and Low Proficiency participants. Treatment coding with the Unaltered condition and Native speakers as the intercept was then used to examine the difference between the High Proficiency and Native speakers.

**Table 2:** **A log mixed-effects model with coefficient estimates, standard errors, Wald's *z-*values, and the significance level for each Time Manipulation condition and Proficiency level**.

	**Estimate**	***SE***	***z*-value**	***p-*value**
(Intercept—*Unaltered*, low proficiency)	1.36	0.42	3.26	***p* < 0.01**
Context expanded	−1.90	0.43	−4.37	***p* < 0.001**
Target compressed	−1.84	0.43	−4.27	***p* < 0.001**
Context compressed	0.46	0.47	0.98	0.33
Target expanded	0.10	0.44	−0.22	0.82
High proficiency	0.95	0.56	1.70	0.09
Native proficiency	1.98	0.70	2.84	***p* < 0.01**
**HIGH PROFICIENCY**				
*Context expanded*	−0.36	0.70	−0.51	0.61
*Target compressed*	0.31	0.70	−0.45	0.66
*Context compressed*	−0.56	0.79	−0.71	0.48
*Target expanded*	−0.56	0.74	−0.76	0.45
**NATIVE**				
*Context expanded*	−2.30	0.82	−2.82	***p* < 0.01**
*Target compressed*	−1.03	0.81	−1.28	0.20062
*Context compressed*	−0.52	1.00	−0.53	0.59883
*Target expanded*	−1.29	0.84	−1.52	0.12750

The Estimates for the Time Manipulation conditions represent the baseline (Low Proficiency).

The results of the mixed effects model analysis show that, as in Experiment 1, the Context Expanded and Target Compressed conditions both lead to a lower likelihood of Long responses (β = −1.90, *z* = −4.37, *p* < 0.001 and β = −1.84, *z* = −4.27, *p* < 0.001). As in Experiment 1, the Context Compressed and Target Expanded conditions did not lead to significantly different likelihood of Long responses than the Unaltered condition. An effect of Proficiency emerged in that Native participants were significantly more likely to report a Long response than Low Proficiency participants (β = 1.98, *z* = 2.84, *p* < 0.01). The difference between High Proficiency participants and Low Proficiency participants was not significant, although High Proficiency participants trended in the direction of reporting more Long responses than Low Proficiency participants (β = 0.95, *z* = 1.70, *p* = 0.09); the difference between High Proficiency and Native participants was not significant (*p* = 0.18). A significant interaction emerged between Proficiency and Time Manipulation condition, in that Native Proficiency participants exhibited lower likelihood of reporting a Long response in precisely the Context Expanded condition, in which the proportion of Long responses would be predicted to be lowered compared to the Unaltered condition (β = −2.30, *z* = 2.82, *p* < 0.01) than the Low Proficiency group. This result may indicate that, despite a higher likelihood of perceiving and reporting the Long response overall, Native participants may be more sensitive to the effect of context speech rate than non-native participants, as they exhibit the predicted effect of a reduction in reporting Long responses when the context speech rate is slowed.

To examine whether there was any independent effect of Rate Type or Locus of Manipulation, as in Experiment 1, only the rate manipulated conditions were included in a model. An ANOVA to test whether Rate Type of Locus themselves, as well as Proficiency, were significant predictors in the model revealed that while neither Rate Type (*p* = 0.34) or Locus (*p* = 0.47) were significant predictors of Long responses, the predicted interaction between these two factors emerged (χ = 102.86, *p* < 0.001), indicating that the Expanded Rate Type for the Context Locus and the Compressed Rate Type for the Target Locus resulted in a lowered proportion of Long responses. Proficiency was also a significant predictor (*p* < 0.05), but there was no interaction between Proficiency and Locus (*p* = 0.60), Proficiency and Rate Type (*p* = 0.26), or between Proficiency, Locus, and Rate Type (*p* = 0.17). These results confirm that with participants of varying proficiency levels in Russian, it is still the conditions in which the Target is relatively short which result in lowered proportions of Long responses.

### Discussion

Experiment 2 provides further evidence that Russian listeners hear more or fewer phonological units (e.g., syllables and/or words) in a critical region of speech depending on the distal context speech rate. The results of Experiment 2 therefore further substantiate the speech rate phenomenon demonstrated in Dilley and Pitt (2010) in a different language, using a distinct paradigm from that of Experiment 1.

Moreover, the expected pattern between Time Manipulation conditions occurred as predicted, indicating that only those conditions in which the Target was relatively short compared with its context (i.e., the Target Compressed and Context Expanded conditions) resulted in altered lexical perception and again confirmed the generalized rate normalization hypothesis. The results suggest that both native and non-native speakers of Russian showed sensitivity to timing information in Russian, so that timing information is available to English speakers for deciphering lexical content in Russian. These results also exhibit a trend suggesting that the ability to use speech rate information in word recognition improves as learners' proficiency in a second language increases. This is evidenced by higher rates of “Long” responses as proficiency increased, implying that more advanced learners could detect the full lexical sequences with greater accuracy than the Low proficiency group.

## General discussion and conclusions

One purpose of this paper was to test two explanations for findings reported by Dilley and Pitt (2010) for English, in which manipulating context speech rate caused listeners to perceive different amounts of phonological material (i.e., syllables or words). Two experiments tested a generalized rate normalization hypothesis against a rate mismatch hypothesis; the former predicted that altered lexical perception would occur only when critical target speech material was made relatively short compared to the context speech rate, while the latter predicted that all conditions in which the speech rate of the critical lexical item and the context were mismatched would yield altered lexical perception. In Experiment 1, native Russian speakers gave a free response to the lexical sequences they heard in each sentence. In Experiment 2, native Russian speakers, as well as native English-speaking learners of Russian, listened to Russian sentences and selected which of two lexical interpretations they heard, where one contained more phonological material than the other.

The results firmly supported the generalized rate normalization account of these effects. The rate of veridical lexical responses significantly decreased only when the duration of the target material was relatively short compared to the rate of the context. The results of the current experiments demonstrate the rapid and seamless integration of signal-based cues (spectral, temporal) and knowledge-based cues (syntactic, semantic) during spoken word recognition.

In addition to confirming that the effect of context speech rate is dependent on the relative length of the target and context portions of the utterance, the results of these experiments confirm that the effects of context speech rate on lexical perception are not restricted to a specific syntactic and prosodic class of items, namely, monosyllabic function words, and that they can occur in another language. Instead, the materials utilized in the current experiments in the Russian language comprised a set of reduced syllables that occurred in a variety of prosodic positions, in addition to consisting of *variable amounts* of phonological material, from syllables consisting of single phonemes within a word to those that comprise a whole monosyllabic function word. This complements prior research showing the effects of speech rate in the identification of a specific phonemes and category boundaries (Miller and Liberman, 1979; Nygaard et al., 1992; Volaitis and Miller, 1992; Sommers et al., 1994). While it has also been shown that temporal information used in phoneme identification can affect speed and accuracy in lexical access, (Davis et al., 2002; Salverda et al., 2003), the present results for Russian, like the previous results for English (Dilley and Pitt, 2010) show that the temporal information from the context speech rate can affect not only the accurate classification of a single phoneme, but the perception of different numbers of phonological units.

Both experiments showed that the speech rate effects first demonstrated by Dilley and Pitt (2010) for English replicate in a new language, Russian; moreover, this is the first research report to focus on perception of casual speech in Russian. Russian listeners heard more or fewer phonological units (e.g., syllables and/or words) in a critical region of speech, depending on the context speech rate. The current findings therefore provide the first replication of the speech rate phenomenon demonstrated in Dilley and Pitt (2010) in a distinct language, suggesting that the effects of speech rate normalization are not a language-specific effect restricted to the English language. Instead, these effects would be expected to occur more generally and apply in a variety of languages and speech contexts.

These results also have implications for the role of speech rate effects in cross-language perception. The issue of how language background affects speech perception and spoken word recognition has been of substantial interest in recent years (Flege et al., 1997; Iverson et al., 2003; Best and Tyler, 2007), but this research has mainly focused on segment perception. In contrast, relatively few studies have addressed the topic of second language learners' perception of more broadly implemented timing and pitch, or prosodic, information; most of these studies have focused largely on word-level stress and accent perception and production (e.g., Flege and Bohn, 1989; Guion et al., 2004; Dupoux et al., 2010). Russian exhibits a number of phonological attributes which differ dramatically from those of English, including distinctive phonotactic restrictions (e.g., /zd/ onset clusters), differences in the phoneme inventory, and a three-way system of vowel reduction (Bondarko, 1998; Padgett and Tabain, 2005). Therefore, Experiment 2 tested English listeners' sensitivity to temporal information in Russian, and the results indicated that both native Russian speakers and learners with varying degrees of proficiency in Russian demonstrated similar patterns of temporal information integration in lexical perception. Even the low proficiency second language listeners were shown to be attuned to timing information in the acoustic signal. Such findings are consistent with the idea that certain global temporal cues could be universally available to language learners. In other words, rate normalization may occur somewhat automatically, even in non-native speech perception, so that learners of a second language are able to apply their native language temporal processing to the non-native language. In this sense, the perception of speech rate and the application of timing information to lexical interpretation, may be an instance where second language perception is not negatively impacted by interference from the native language. In contrast, differences in first and second language phoneme categories and phonotactics, in particular, have been shown to result in altered perceptions of phoneme sequences in a non-native language. This has been demonstrated in numerous cross-linguistic environments, including English listeners' perception of Slavic languages (Flege et al., 1997; Davidson, 2007). Therefore, transfer effects for the incorporation of speech rate information into lexical access cannot be considered entirely predictable, and it was not at all clear that native language strategies for incorporating rate information would necessarily lead to native-like perception patterns in a second language.

The results of Experiment 2, which indicate an effect of second language proficiency on perception patterns, cannot be exclusively explained by the transfer of knowledge of native language temporal patterns, or a universally available mechanism for temporal information processing in speech. Instead, these findings suggest that the ability to use temporal cues in non-native lexical perception improves with increased proficiency in the second language. In general, non-native listeners have difficulties with word segmentation, especially in cases where allophonic cues, such as segmental duration, are needed to perceive a word boundary location (e.g., Altenberg, 2005; Ito and Strange, 2009). Non-native listeners can show an increased reliance on acoustic cues when lexical-semantic information is compromised in the signal, relying less on lexical information than native speakers do (Bradlow and Alexander, 2007; Mattys et al., 2010). The results of the current experiments show that, even though learners of Russian may not use temporal information as effectively as native speakers, they were able to fairly successfully integrate context speech rate information in lexical access. As discussed above, it is possible that interference from differences between the phoneme inventories and phonotactics of the native and non-native languages, or certain phonological processes such as vowel reduction, may have affected the general accuracy with which learners identified phonological material. Therefore, with increased experience in the second language, non-native listeners appeared better able to integrate different types of information across utterances, including temporal information, resulting in more native-like response patterns.

Another finding from the current experiments concerns the locus of the speech rate manipulations; in all conditions, the rate mismatches occurred at locations non-adjacent to the phonological material subject to altered perceptions. In certain cases, where the target material consisted of a whole, monosyllabic function word, lexical recognition was affected by temporal information occurring in disparate lexical items. This contrasts with previous work showing speech rate minimally affects segmental processing in material non-adjacent to a critical segment of experimental interest (Miller and Liberman, 1979; Summerfield, 1981; Newman and Sawusch, 1996). A separate line of research has investigated the effects of distal pitch and timing information on the perception of word boundary locations occurring later in an utterance (Dilley and McAuley, 2008; Dilley et al., 2010; Brown et al., 2011). In those studies, the perception of patterns of strong and weak syllables in the distal context affected the way in which listeners grouped syllables into words. Whether these various speech rate and other distal processing effects stem from the same or different mechanisms will be a topic for further research.

Overall, these results indicate that speech rate and duration information may play essential roles in lexical perception and recognition in speech and that these effects are not language-specific. In addition, the results provide an explanation for the nature of the speech rate effects by supporting a generalized rate normalization account of effects observed across two languages, English and Russian. Listeners compare the duration of a portion of speech material with the surrounding context, and use the relative duration of a target sequence to aid in lexical recognition. In cases of potentially ambiguous spectral cues to lexical content, such as those associated with casual speech, context speech rate information may be critical to accurate lexical perception and segmentation of the speech signal. The present results also demonstrate that distal temporal information is used by both native and non-native speakers in lexical recognition in Russian. Together with the findings of Dilley and Pitt (2010) for English, the present findings suggest that the basic mechanisms involved in the use of temporal information in speech perception may be part of the general architecture of auditory perception.

## Authors contributions

Author Laura C. Dilley was responsible for conceptualization and design of the experiments and contributed to writing of the manuscript. Author Tuuli H. Morrill contributed to data analysis and writing of the manuscript. Author Elina Banzina contributed to stimulus construction, data collection, data analysis, and writing of the manuscript.

### Conflict of interest statement

The authors declare that the research was conducted in the absence of any commercial or financial relationships that could be construed as a potential conflict of interest.

## Figures and Tables

**Table A1:**
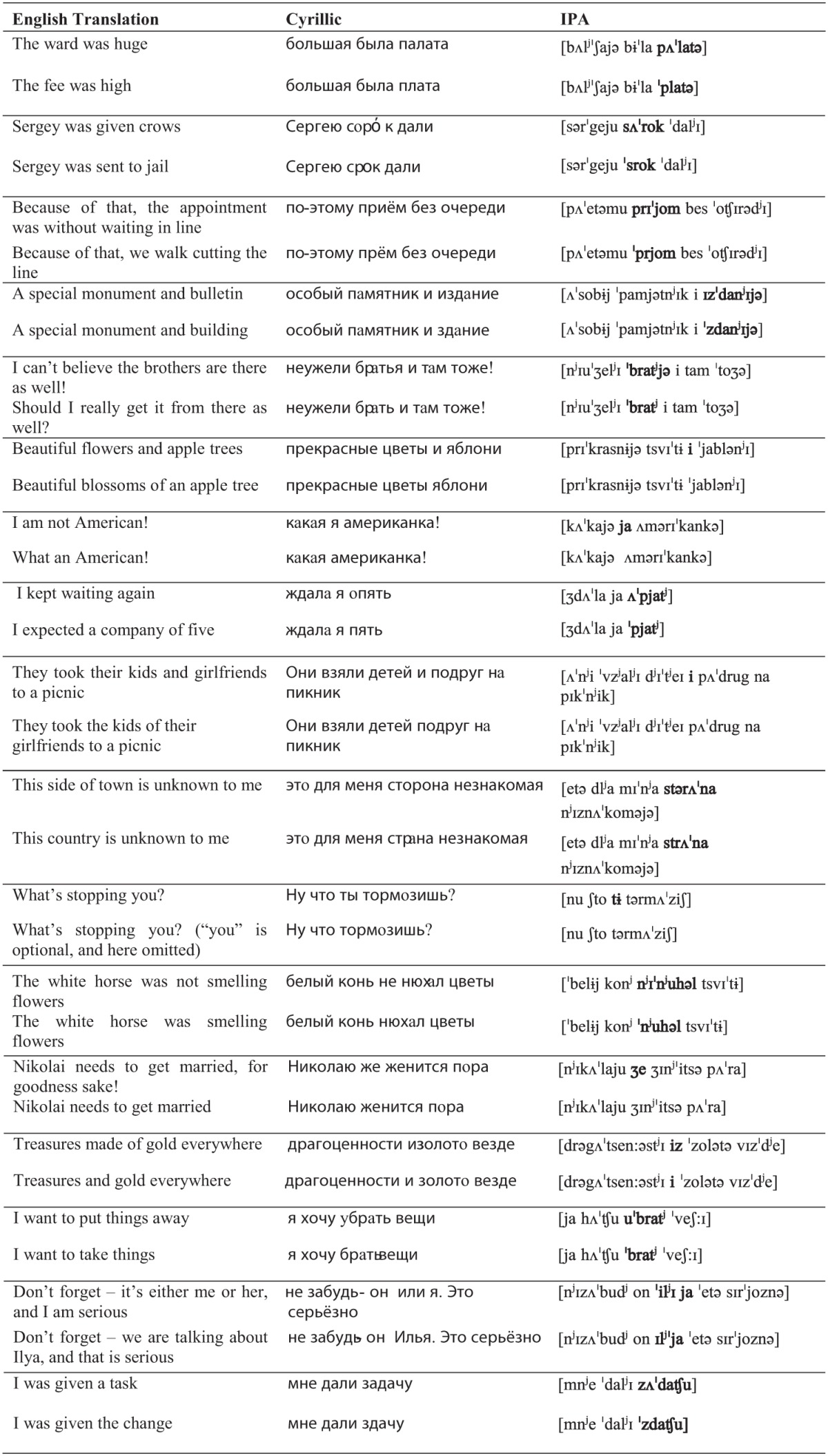
**Stimulus sentences**.
